# Supplementary material for: Youth partnership in suicide prevention research: moving beyond the safety discourse
Source: BMJ Open. 2023 Oct 27;13(10):e076885. doi: 10.1136/bmjopen-2023-076885 (PMC10619073; doi:10.1136/bmjopen-2023-076885)
Supplement: Author's manuscript [file bmjopen-2023-076885.draft_revisions.pdf]

BMJ Open

BMJ Open is committed to open peer review. As part of this commitment we make the peer review history of every article we publish publicly available.

When an article is published we post the peer reviewers' comments and the authors' responses online. We also post the versions of the paper that were used during peer review. These are the versions that the peer review comments apply to.

The versions of the paper that follow are the versions that were submitted during the peer review process. They are not the versions of record or the final published versions. They should not be cited or distributed as the published version of this manuscript.

BMJ Open is an open access journal and the full, final, typeset and author-corrected version of record of the manuscript is available on our site with no access controls, subscription charges or pay-per-view fees (http://bmjopen.bmj.com).

If you have any questions on BMJ Open's open peer review process please email info.bmjopen@bmj.com

BMJ Open

Youth partnership in suicide prevention research: moving beyond the safety discourse

Journal:	*BMJ Open*
Manuscript ID	bmjopen-2023-076885.R1
Article Type:	Communication
Date Submitted by the Author:	05-Jul-2023
Complete List of Authors:	Michail, Maria; University of Birmingham, Institute for Mental Health, School of Psychology<br>Morgan, Jamie; University of Birmingham, Institute for Mental Health, School of Psychology<br>Lavis, Anna; University of Birmingham, Institute of Applied Health Research
**Primary Subject Heading**:	Mental health
Secondary Subject Heading:	Ethics
Keywords:	Patient Participation, Suicide & self-harm < PSYCHIATRY, MENTAL HEALTH

SCHOLARONE™
Manuscripts

I, the Submitting Author has the right to grant and does grant on behalf of all authors of the Work (as defined in the below author licence), an exclusive licence and/or a non-exclusive licence for contributions from authors who are: i) UK Crown employees; ii) where BMJ has agreed a CC-BY licence shall apply, and/or iii) in accordance with the terms applicable for US Federal Government officers or employees acting as part of their official duties; on a worldwide, perpetual, irrevocable, royalty-free basis to BMJ Publishing Group Ltd ("BMJ") its licensees and where the relevant Journal is co-owned by BMJ to the co-owners of the Journal, to publish the Work in BMJ Open and any other BMJ products and to exploit all rights, as set out in our licence.

The Submitting Author accepts and understands that any supply made under these terms is made by BMJ to the Submitting Author unless you are acting as an employee on behalf of your employer or a postgraduate student of an affiliated institution which is paying any applicable article publishing charge ("APC") for Open Access articles. Where the Submitting Author wishes to make the Work available on an Open Access basis (and intends to pay the relevant APC), the terms of reuse of such Open Access shall be governed by a Creative Commons licence – details of these licences and which Creative Commons licence will apply to this Work are set out in our licence referred to above.

Other than as permitted in any relevant BMJ Author's Self Archiving Policies, I confirm this Work has not been accepted for publication elsewhere, is not being considered for publication elsewhere and does not duplicate material already published. I confirm all authors consent to publication of this Work and authorise the granting of this licence.

For peer review only

Youth partnership in suicide prevention research: moving beyond the safety discourse

Maria Michail[1*] PhD, Jamie Morgan[1] MA, Anna Lavis[2] PhD

[1] Institute for Mental Health, School of Psychology, University of Birmingham, B15 2TT, UK

[2] Institute of Applied Health Research, University of Birmingham, Birmingham, B15 2TT, UK

***Corresponding author**

Dr Maria Michail
Institute for Mental Health,
School of Psychology,
University of Birmingham,
B15 2TT,
UK
Email: m.michail@bham.ac.uk

For peer review only

Abstract

In this Communication article, we discuss co-production in suicide prevention research, with an emphasis on involving young people. We critically reflect on the lessons we have learned by working alongside young people, and how these lessons may be useful to other research teams.

The meaningful involvement of young people in the design, implementation and translation of mental health research has received significant attention over the last decade. For most funding bodies, the involvement of patients and the public in the planning and delivery of research is advised and, in many cases, mandatory. When it comes to suicide prevention research, however, things are slightly different in practice. Involvement of young people in suicide prevention research has often been considered a controversial, unfeasible, and even risky endeavour. In our experiences of working in this field, such concerns are expressed by funders, Higher Education Health and Safety committees and practitioners.

By presenting an example from our research where the involvement of young people as experts by experience was integral, we highlight key lessons learnt that could maximise the potential of youth partnership in suicide prevention research. These lessons take on particular importance in mental health research against the background of long-entrenched power differences and the silencing of service user voices. Professional knowledge, obtained through education and vocational training, has historically taken priority over experiential knowledge obtained through lived experience, in psychiatric practice and research. Although this hierarchy has widely been challenged, any account of co-production in mental health research is positioned against that background, and the remnants of those

For peer review only - http://bmjopen.bmj.com/site/about/guidelines.xhtml

inequitable power relationships arguably take on greater resonance in suicide prevention research and require careful consideration to ensure meaningful involvement.

We conclude that progress in suicide research cannot be fulfilled without the meaningful involvement of, and partnership with, young people with lived experience.

Introduction

Involvement, co-production, co-design – Does terminology matter?

Involvement has been described as "an evolving concept in the mental healthcare literature"[1] and is a term that encompasses service user involvement in both mental healthcare and research. In terms of the former, moves towards involving service users in their own care and decision making ensued from both deinstitutionalisation and the political and philosophical critiques of psychiatry that emerged through the 1960s and 1970s in Europe and North America. These moves challenged both widespread institutionalisation and the biomedical model of mental illness. Service user involvement is now widely valued in health and social care.[2] It is key across a range of areas, from an individual's own care and decision making, to the service design, delivery and evaluation, policymaking, and research across a range of settings and topic areas. *No research about us without us* is a phrase commonly used to highlight the need for youth voices to be at the front and centre of research that has the potential to affect them, their families, and the services or treatment they might receive, and is formalised in INVOLVE's[3] definition of involvement as "research being carried out 'with' or 'by' non-professionals rather than 'to', 'about' or 'for' them."

Against this background, the terms co-production and co-design in applied health research are often used interchangeably, but it is key to note that both fall at one end of what has been termed a continuum of involvement.[4] At one end of this continuum is "*tokenism when a few service users might be asked to comment on or react to an agenda, project or document which has already been developed"* (ibid.). The other end of the continuum is "*characterised by a different approach and ideology in which clients' expertise and knowledge is valued and an alternative discourse is recognised where service users may be involved in their own organisations in which they support each other and promote their shared agenda*" (ibid.). This latter is underpinned by an emphasis on the value of experiential knowledge, placing this on an equal footing with professional knowledge. In relation to the history of silencing and disempowerment of mental health service users, noted above, this "*can be seen as an explicit call for persons with mental health diagnoses to exercise their rights as citizens to participate in the development, execution, and outcomes of research that directly affects them*"[5], and it is this that has led service user involvement to be described as an "*ethical imperative*".[6] Both co-production and co-design lie at this latter end of the continuum of service user involvement.

The term *co-production* was initially conceptualised in the 1970s by Nobel Prize Winner Professor Elinor Ostrom[7], a political scientist and economist. Ostrom used the term co-production, within the context of public service, to highlight the lack of recognition of the

For peer review only - http://bmjopen.bmj.com/site/about/guidelines.xhtml

contribution of consumers in public service delivery; and the impact this could have on the efficient running of public services. The term co-production has since been used in many sectors including economics;[8] health policy and practice[9] and social care.[10] Within the context of mental health research, co-production goes beyond mere 'collaboration'[11] and it is not simply something that is 'done,' to be implemented or added on; rather it should run throughout the full course of the research, actively shaping how this is done. Co-production is a collaborative model of research, which emphasises the equality between, and combining of, different skills and experiences.[5]

Co-design is linked more closely to the concepts of user engagement and participatory design for the purposes of developing a new product, initiative, or service.[12]

Many attempts have been made to demarcate the terms co-production and co-design, as well as involvement more broadly, but one could ask "*Does it really matter*?". A systematic review[13] of the varied definitions surrounding these terms concluded that these terms are conceptualised in many different ways; and instead of focusing our efforts on finding a common language what we should be focusing on is defining the key principles and values underlying these terms; and how these could be meaningfully translated in every practice. In this Communication article, we will use the terms involvement and co-production.

Youth involvement in mental health research

Within the context of mental health research, the meaningful involvement of young people with lived or living experience in planning, delivering, and implementing research has received widespread attention. The significant benefits resulting from partnering with young people to collaboratively develop and deliver high quality mental health research[14,15] have been widely documented.

It is key not to ignore the potential pitfalls of co-production, such as various risks to both researchers and experts by experience[16] but also to recognise how current limitations may arise from existing contextual inequalities in academia and more broadly.[11] Against the background of the historically embedded power differentials noted above, the possibility of reinforcing such unequal power relations across the various stakeholders needs recognising.[17,18] However, it is also clear that for young people with lived or living experience, co-production can be empowering in that it can offer a way to make a difference, and even to reflect on one's own experiences. [19,20]

Youth involvement in suicide prevention research

Then why is it that when it comes to suicide prevention research, the involvement of young people is often considered risky and unfeasible?

There is, firstly, a pervasive concern about the possibility of triggering distress, and even suicidal thoughts and feelings. This is mirrored by a similar concern when young people take part as research participants too – and yet, taking part in research does not inflict harm and young people cite important benefits.[21] So, why do we hold onto this concern in such a way that it acts to preclude young people from being involved in suicide prevention research? The safety of the young people is of paramount importance and the risk of involvement

For peer review only - http://bmjopen.bmj.com/site/about/guidelines.xhtml

being triggering needs to be acknowledged. It may be that there are limitations to involvement posed by legitimate safeguarding, depending on the topic of the research. However, we argue that the greatest risk to young people is not being heard and included. We risk leaning too heavily on a safety discourse in a way that allows us to not collaboratively find ways to undertake co-production ethically and safely. We also run the risk of being unethical, exclusionary, and paternalistic. These specific risks that arise from, or perhaps rather are *assumed to* arise from, suicide prevention research, show the need to be mindful of not reproducing entrenched power imbalances between service users on the one hand and 'professionals' on the other.

To undertake meaningful involvement in suicide prevention research, there is a need across the team – both young people and researchers – to sit with a certain level of risk. It is key that this is acknowledged at the outset of the research and collaboratively discussed. Before this stage, however, it is also necessary to rethink the way we approach risk in drawing the team together. There is a tendency in even the most ethical and equitable involvement to nevertheless exclude people who may be, or have recently been, suicidal. Given the subject matter and the need to ensure that the voices of young people who are or have been suicidal are placed central to our research praxis, we question whether this is ethical.

The need, therefore, to reflect critically on who is 'allowed' to become involved mirrors who is 'allowed' to take part in suicide prevention research as a participant too. In both contexts, if 'we' as the researchers set the bar in a way that is too risk averse, we will perpetuate the historical power differentials in mental health research and services noted above, and ignore, exclude, and disempower people whose voices should be listened to.

We provide below an example of co-production in suicide prevention research drawn from our own research programme; highlighting opportunities for maximising the potential of partnerships between researchers and young people with lived or living experience. The case study is written by the second author (JM), a member of the Youth Advisory Group (YAG), at the Institute for Mental Health, University of Birmingham. The YAG consists of 18 young people aged between 18-25 with experience of or a strong interest in youth mental health, who work collaboratively with researchers to create and shape research into youth mental health including self-harm and suicide.

Case study

#MyGPguide - Visiting your general practitioner: a guide for young people with lived experience of self-harm and suicidality

#MyGPguide[22] is an evidence-based resource co-designed with young people for young people with lived experience of self-harm and/or suicidal behaviour to prepare them for their consultation with their General Practitioner (GP) offering practical support and guidance on: i) What to consider before they visit their doctor, including preparing questions and booking an appointment; ii) How to manage the consultation, what their rights are with respect to confidentiality, what questions their doctor might ask them; and how to manage discussions about medication, safety planning and referral to mental health

For peer review only - http://bmjopen.bmj.com/site/about/guidelines.xhtml

services; iii) What to do after the consultation and how their doctor can support them including signposting and accessing professional support.

Consultations about self-harm and suicidal thoughts and feelings can be challenging for both GPs and young people[23,24]. *#MyGPguide*, offers evidence-based, accessible, and practical tips to facilitate the best consultation and support for young people at-risk of suicide.

This account of the creation of #MyGPguide will highlight and reflect on various issues that have been raised when considering the feasibility, value, ethics and safeguarding of co-production in suicide prevention research.

Over a period of six months, six members from the Institute of Mental Health, University of Birmingham YAG worked in partnership with the project lead (first author) and the Institute's Youth Participation Lead (YPL) to co-design *#MyGPguide*. The role of the YPL is to facilitate meetings between researchers and the YAG; to process requests from researchers to involve the YAG in their work, and likewise help make it easy and comfortable for the YAG to be involved. With regards to safety, the YPL's role is important as they provide a constant for the YAG in their meetings with different researchers. By being a friendly face, and someone who is specifically there to facilitate the work of the YAG, the feelings that might accompany a young person in this sort of environment are effectively mitigated.

Consultations took place online due to COVID-19 restrictions at the time. The first consultation started with the YPL reminding everyone of the YAG terms of reference; and the project lead sharing key ground rules that would ensure that young people were involved in a safe, inclusive, and respectful way throughout the process. The ground rules focused on i) highlighting the importance of confidentiality (and its limits) and anonymity; ii) acknowledging and respecting different and complementary types of expertise; iii) listening respectfully to one another; iv) allowing others the time and space to share their story; v) taking turns in speaking and being mindful of dominating discussions.

The first stage of this project was an 'initial ideas' meeting which involved brainstorming and discussing initial thoughts about the content and overall direction that YAG members wanted the guide to take. This was a productive meeting and young people were given multiple chances to share their views. It is important to note that from the beginning and throughout the whole project young people were never asked to share or disclose personal experiences or events that might have been relevant to the project. Instead, young people were encouraged to draw upon their own experience to meaningfully inform the content of the guide. Young people were encouraged to contribute in different ways depending on their preference. This included sharing their views during the online consultations; writing down thoughts and emailing them to the project lead; or having a one-to-one chat with the project lead. For me, this flexible approach was invaluable as I found it difficult to share in the group some of the points that I wanted to make. These points related to my own and my friends' experience of visiting the GP and how I wanted these points conveyed in *#MyGPguide*. In response, the project lead expressed the importance of these points and their added value to *#MyGPguide*. The project lead subsequently described how these

For peer review only - http://bmjopen.bmj.com/site/about/guidelines.xhtml

points would be incorporated in the guide ensuring that this process was carried out in a way that would accurately reflect my contribution i.e., staying true to my voice.

Following the initial brainstorming session, the first draft of *#MyGPguide* was circulated to all YAG members involved in the project and we were encouraged to read the guide and send our feedback. Feedback could be given via different ways including highlighting or using track changes on the document, or simply drafting the feedback and emailing this back to the project lead. It was clear where our comments had been included in the draft guide alongside the evidence-base.

To bring the guide to life, the project lead offered YAG members the opportunity to be involved in a few other ways. These included producing a quote relating to a specific aspect of the guide (e.g., the importance of confidentiality) to be embedded within the guide, involvement in a video that would accompany the guide, and an opportunity to be involved in a webinar to promote *#MyGPguide*. Each of these opportunities were once again accompanied by the flexibility and inclusivity which characterised the whole process of involvement.

Subsequent iterations of *#MyGPguide* were shared with the YAG, and members were always encouraged to make additional contributions and provide feedback. It was clearly demonstrated to young people how their contributions had informed the content and design of *#MyGPguide* as well as a dissemination plan targeting diverse audiences including young people, families, primary care practitioners and schools. YAG members were kept informed throughout the project about timelines, milestones including the release of *#MyGPguide*; and supported to take part in dissemination and outreach activities to promote the guide.

Reflecting on my own experience of taking part in the design of *#MyGPguide*, neither I nor any other young person were ever asked to reveal personal information, and any that was given was given voluntarily, without prompt and in line with the YAG terms of reference. The discussions, either as part of the group or one-to-one, were equitable and balanced. The way young people's contributions influenced *#MyGPguide* was clearly demonstrated to us; and where feedback had not been taken on board the rationale was provided. For example, although important, some points and ideas might not have fit the form the guide was aiming to take, could not offer additional value, or did not speak to the target audience.

At no point did I feel like my contribution would make or break the guide and I was reassured that I could withdraw at any time. Crucially, though, YAG members were consistently reminded of how their involvement and any contribution they did make was important and valued.

Furthermore, the flexibility described in this account was one of the most central themes that ensured safety in the involvement of YAG. Although extremely important, safety does not simply refer to the idea of keeping someone safe from physical or psychological harm. The term safety within the context of suicide prevention research should also be taken to mean how comfortable and able a young person is to fully draw upon their experience to inform the research they are involved in. The flexibility of involvement here was crucial to

For peer review only - http://bmjopen.bmj.com/site/about/guidelines.xhtml

maintaining a comfortable environment. Indeed, if young people do not feel safe or comfortable, they will not be able to fully apply their experiences and expertise or they will avoid participating altogether. It is therefore in both the young persons and the researchers' interests to ensure safety during involvement.

Key lessons for youth involvement in suicide prevention research

Young people were at the forefront of designing and disseminating *#MyGPguide*. Using the case study above and our previous research experience and experiential knowledge, we draw together key lessons underlying the optimal involvement of young people in suicide-related research.

Firstly, having clearly defined and mutually agreed terms of reference and ground rules helps generate and sustain inclusivity throughout the process of youth involvement in suicide prevention research. As Clarke et al[25] highlighted, inclusivity is not simply about bringing together a group of stakeholders to share and exchange knowledge and views. Inclusivity is about generating and sustaining mutually agreed goals; sharing the same vision, or, as Clarke et al[25] described it "*a shared sense of identity and solidarity*".

Secondly, consulting with young people about potential ethical considerations, safety issues or triggers before and during co-production and putting in place safety protocols tailored to young people's needs could ensure their safe involvement. Such protocols could take the form of a wellness or safety plan where researchers and young people work together to identify potential triggers, strategies that the young person can use when they are feeling upset, a support person the researchers could contact if the young person wishes so. Having a one-to-one debriefing session with young people would also offer them the opportunity to highlight things that worked well for them during a co-production session and things that could be improved in future sessions. We cannot eliminate risk, but we can put in place robust processes that would help researchers mitigate risk. It is crucial to recognise that young people can draw upon their own experiences to inform research (and through this practice and policy) without necessarily having to disclose or share those experiences. It is important to set the parameters of sharing at the outset of any research, both creating a safe space to do so and also emphasising that this is not an expectation. Having a dedicated youth participation lead to support both young people and researchers during this process in a safe, productive, and ethically sound way is key.

Thirdly, setting a culture of open, transparent, and reciprocal communication between young people and researchers is key to the establishment of an equal relationship among all those involved in the co-production and the continual challenging of hierarchies. Co-production should be a professional relationship between young people and researchers and as in any other type of professional relationship, there should be clearly defined roles, clear objectives, and an expectation of being listened to and valued.

It is our ethical and moral imperative, as researchers, to create a safe and inclusive environment that would empower the involvement of young people with lived or living experience of self-harm and suicidal behaviour in research that affects them, their care and

For peer review only - http://bmjopen.bmj.com/site/about/guidelines.xhtml

quality of life. Progress in the field of suicide prevention cannot be achieved without the meaningful involvement of young people.

Contributors

MM, AL and JM conceptualised the paper. MM wrote the first draft of the manuscript. All authors were involved in further revisions and writing of the final version.

Declaration of interests

None declared. *#MyGPguide* is a non-commercial product for which the authors receive no royalties.

Role of funding source

The authors did not receive any funding for this manuscript.

REFERENCES

1. Millar, S.L., Chambers, M. and Giles, M. (2016), Service user involvement in mental health care: an evolutionary concept analysis. *Health Expect* 2016; **19**: 209-221.
2. Fenton, S-J., Carr, S., Boyden, L. Molloy, J., Moyse, H., Rathore, I., Skinner, B., Tresadern, C., Virgo, H., Willams, B. and Campbell, N. Valuing Youth Involvement in Mental Health Service Design and Delivery In Theodosiou, L., Knightsmith, P., Paula, P. and Bailey, S. (eds.) Children and Young People's Mental Health London: Pavilion pp. 163-178, 2020
3. INVOLVE. Briefing Notes for Researchers: Public Involvement in NHS, Public Health and Social Care Research, 2012 (page 7).
4. Henwood M. Involvement of service users in social care research. Disability, 2007 https://www.communitycare.co.uk/2007/09/12/involvement-of-service-users-in-social-care-research/ (Accessed 26 May 2023)
5. Lignou S, Capitao L, Hamer-Hunt J.M., Singh I. Co-Production: An Ethical Model for Mental Health Research? *Am J Bioeth* 2019; **19**:8, 49-51
6. Rose, D. Patient and public involvement in health research: Ethical imperative and/or radical challenge? *J Health Psychol* 2014; **19**: 1, 149-158
7. Ostrom E, Parks RB. Suburban police departments: Too many and too small? Department of Political Science, Indiana University; 1973.
8. Stephens L., Josh Ryan-Collins J., Boyle E.D. Co-production. A Manifesto for growing the core economy. New Economics Foundation. London, 2008 https://neweconomics.org/uploads/files/5abec531b2a775dc8d_qjm6bqzpt.pdf (Accessed 26 May 2023)

For peer review only - http://bmjopen.bmj.com/site/about/guidelines.xhtml

9. Coote A. Claiming the health dividend: unlocking the benefits of NHS spending. London: King's Fund, 2002 Claiming the Health Dividend: Unlocking the benefits of NHS spending, Summary - Anna Coote (ed) - The King's Fund, 1st May 2002 (kingsfund.org.uk) (Accessed 26 May 2023)

10. Needham C., Carr S. SCIE research briefing 31: co-production: an emerging evidence base for adult social care transformation. Social Care Institute for Excellence, London, 2019 https://lx.iriss.org.uk/sites/default/files/resources/briefing31.pdf (Accessed 26 May 2023)

11. Williams, O., Sarre, S., Papoulias, Knowles S., Robert G., Beresford P., Rose D., Carr S., Kaur M., Palmer V. Lost in the shadows : reflections on the dark side of co-production. *Health Res Policy Sys* 2020; **18**:1, 43

12. Vargas C, Whelan J, Brimblecombe J, Allender S. Co-creation, co-design and co-production for public health: a perspective on definitions and distinctions. *Public Health Res* Pract 2022; **32**:2, e3222211.

13. Masterson, D, Areskoug Josefsson, K, Robert, G, Nylander, E, Kjellström, S. Mapping definitions of co-production and co-design in health and social care: a systematic scoping review providing lessons for the future. *Health Expect* 2022; **25**: 902- 913

14. Ennis L, Wykes T. Impact of patient involvement in mental health research: longitudinal study. *Br J Psychiatry* 2013; **203**:5, 381-386.

15. Staley K. A series of case studies illustrating the impact of service user and care involvement on research. London, UK: Mental Health Research Network; 2013

16. Oliver K, Kothari A, Mays N. The dark side of coproduction: do the costs outweigh the benefits for health research? *Health Res Policy Syst* 2019; **17**:1. 33.

17. Carr S. Can Co-production Really Transform UK Mental Health Services? Mad in America, 2016. https://www.madinamerica.com/2016/08/can-co-production-really-transform-uk-mental-health-services/ (Accessed 26 May 2023)

18. Daya, I., Hamilton, B. and Roper, C. Authentic engagement: A conceptual model for welcoming diverse and challenging consumer and survivor views in mental health research, policy, and practice. *Int J Mental Health Nurs* 2020; **29**:2, 299-311.

19. Awenat YF, Moore C, Gooding PA, Ulph F, Mirza A, Pratt D. Improving the quality of prison research: a qualitative study of ex-offender service user involvement in prison suicide prevention research. *Health Expect* 2018; **21**:1, 100-109.

20. MacLean S, MacKie C, Hatcher S. Involving people with lived experience in research on suicide prevention. *CMAJ* 2018; **190**(Suppl), S13-S14.

21. Lockwood, J., Townsend, E., Royes, L. *et al.* What do young adolescents think about taking part in longitudinal self-harm research? Findings from a school-based study. *Child Adolesc Psychiatry Ment Health* 2018; **12**: 23

22. Michail M., Rowmell H., Mitchell L., Morgan J., Rathore I., Reid K., Tresadern C. et al. *#MyGPguide* - Visiting your General Practitioner: A guide for young people with lived experience of self-harm and suicidality. Birmingham: Institute for Mental Health,

For peer review only - http://bmjopen.bmj.com/site/about/guidelines.xhtml

School of Psychology, University of Birmingham, 2021 https://doi.org/10.6084/m9.figshare.17069660.v1 (Accessed 26 May 2023)

23. Michail M, Tait L. Exploring general practitioners' views and experiences on suicide risk assessment and management of young people in primary care: a qualitative study in the UK. *BMJ Open* 2016; **6**: e009654
24. Farr J, Surtees ADR, Richardson H, Michail M. Exploring the processes involved in seeking help from a General Practitioner for young people who have been at risk of suicide. *Int J Environ Res Public Health* 2021; **18**:4, 2120.
25. Clarke, J, Waring, J, Timmons, S. The challenge of inclusive coproduction: The importance of situated rituals and emotional inclusivity in the coproduction of health research projects. *Soc Policy Admin* 2019; **53**: 233– 248

For peer review only

For peer review only - http://bmjopen.bmj.com/site/about/guidelines.xhtml

BMJ Open

Youth partnership in suicide prevention research: moving beyond the safety discourse

Journal:	*BMJ Open*
Manuscript ID	bmjopen-2023-076885.R2
Article Type:	Communication
Date Submitted by the Author:	20-Sep-2023
Complete List of Authors:	Michail, Maria; University of Birmingham, Institute for Mental Health, School of Psychology<br>Morgan, Jamie; University of Birmingham, Institute for Mental Health, School of Psychology<br>Lavis, Anna; University of Birmingham, Institute of Applied Health Research
**Primary Subject Heading**:	Mental health
Secondary Subject Heading:	Ethics
Keywords:	Patient Participation, Suicide & self-harm < PSYCHIATRY, MENTAL HEALTH

SCHOLARONE™
Manuscripts

I, the Submitting Author has the right to grant and does grant on behalf of all authors of the Work (as defined in the below author licence), an exclusive licence and/or a non-exclusive licence for contributions from authors who are: i) UK Crown employees; ii) where BMJ has agreed a CC-BY licence shall apply, and/or iii) in accordance with the terms applicable for US Federal Government officers or employees acting as part of their official duties; on a worldwide, perpetual, irrevocable, royalty-free basis to BMJ Publishing Group Ltd ("BMJ") its licensees and where the relevant Journal is co-owned by BMJ to the co-owners of the Journal, to publish the Work in BMJ Open and any other BMJ products and to exploit all rights, as set out in our licence.

The Submitting Author accepts and understands that any supply made under these terms is made by BMJ to the Submitting Author unless you are acting as an employee on behalf of your employer or a postgraduate student of an affiliated institution which is paying any applicable article publishing charge ("APC") for Open Access articles. Where the Submitting Author wishes to make the Work available on an Open Access basis (and intends to pay the relevant APC), the terms of reuse of such Open Access shall be governed by a Creative Commons licence – details of these licences and which Creative Commons licence will apply to this Work are set out in our licence referred to above.

Other than as permitted in any relevant BMJ Author's Self Archiving Policies, I confirm this Work has not been accepted for publication elsewhere, is not being considered for publication elsewhere and does not duplicate material already published. I confirm all authors consent to publication of this Work and authorise the granting of this licence.

For peer review only

Youth partnership in suicide prevention research: moving beyond the safety discourse

Maria Michail[1*] PhD, Jamie Morgan[1] MA, Anna Lavis[2] PhD

[1] Institute for Mental Health, School of Psychology, University of Birmingham, Birmingham B15 2TT, UK

[2] Institute of Applied Health Research, Medical and Dental Sciences, University of Birmingham, Birmingham B15 2TT, UK

***Correspondence to:**

Dr Maria Michail
Institute for Mental Health, School of Psychology, University of Birmingham, Birmingham B15 2TT, UK
Email: m.michail@bham.ac.uk

For peer review only

For peer review only - http://bmjopen.bmj.com/site/about/guidelines.xhtml

Abstract

In this Communication article, we discuss co-production in suicide prevention research, with an emphasis on involving young people. We critically reflect on the lessons we have learned by working alongside young people, and how these lessons may be useful to other research teams.

The meaningful involvement of young people in the design, implementation and translation of mental health research has received significant attention over the last decade. For most funding bodies, the involvement of patients and the public in the planning and delivery of research is advised and, in many cases, mandatory. When it comes to suicide prevention research, however, things are slightly different in practice. Involvement of young people in suicide prevention research has often been considered a controversial, unfeasible, and even risky endeavour. In our experiences of working in this field, such concerns are expressed by funders, Higher Education Health and Safety committees and practitioners.

By presenting an example from our research where the involvement of young people as experts by experience was integral, we highlight key lessons learnt that could maximise the potential of youth partnership in suicide prevention research. These lessons take on particular importance in mental health research against the background of long-entrenched power differences and the silencing of service user voices. Professional knowledge, obtained through education and vocational training, has historically taken priority over experiential knowledge obtained through lived experience, in psychiatric practice and research. Although this hierarchy has widely been challenged, any account of co-production in mental health research is positioned against that background, and the remnants of those inequitable power relationships arguably take on greater resonance in suicide prevention research and require careful consideration to ensure meaningful involvement.

We conclude that progress in suicide research cannot be fulfilled without the meaningful involvement of, and partnership with, young people with lived experience.

Introduction

Involvement, co-production, co-design – does terminology matter?

Involvement has been described as "an evolving concept in the mental healthcare literature" (1) and is a term that encompasses service user involvement in both mental healthcare and research. In terms of the former, moves towards involving service users in their own care and decision making ensued from both deinstitutionalisation and the political and philosophical critiques of psychiatry that emerged through the 1960s and 1970s in Europe and North America. These moves challenged both widespread institutionalisation and the biomedical model of mental illness. Service user involvement is now widely valued in health and social care (2). It is key across a range of areas, from an individual's own care and decision making, to service design, delivery and evaluation, policymaking, and research across a range of settings and topic areas. *No research about us without us* is a phrase commonly used to highlight the need for youth voices to be at the front and centre of research that has the potential to affect them, their families, and the services or treatment

For peer review only - http://bmjopen.bmj.com/site/about/guidelines.xhtml

they might receive, and is formalised in INVOLVE's (3) definition of involvement as "research being carried out 'with' or 'by' non-professionals rather than 'to', 'about' or 'for' them."

Against this background, the terms co-production and co-design in applied health research are often used interchangeably, but it is key to note that both fall at one end of what has been termed a continuum of involvement (4). At one end of this continuum is "*tokenism when a few service users might be asked to comment on or react to an agenda, project or document which has already been developed*" (ibid.). The other end of the continuum is "*characterised by a different approach and ideology in which clients' expertise and knowledge is valued and an alternative discourse is recognised where service users may be involved in their own organisations in which they support each other and promote their shared agenda*" (ibid.). This latter is where co-production and co-design fall, and is underpinned by an emphasis on the value of experiential knowledge, recognising this as being on an equal footing with professional knowledge. Given the history of silencing and disempowerment of mental health service users that is now widely acknowledged, this "*can be seen as an explicit call for persons with mental health diagnoses to exercise their rights as citizens to participate in the development, execution, and outcomes of research that directly affects them*" (5); and it is this that has led service user involvement to be described as an "*ethical imperative*" (6).

The term *co-production* was initially conceptualised in the 1970s by Nobel Prize Winner Professor Elinor Ostrom (7), a political scientist and economist. Ostrom used the term co-production, within the context of public services, to highlight the lack of recognition of the contribution of consumers in public service delivery, and the impact this could have on the efficient running of public services. The term co-production has since been used in many sectors including economics (8); health policy and practice (9) and social care (10). Within the context of mental health research, co-production goes beyond mere 'collaboration' (11) and it is not simply something that is 'done,' to be implemented or added on; rather it should run throughout the full course of the research, actively shaping how this is done. Co-production is a collaborative model of research, which emphasises the equality between, and combining of, different skills and experiences.[5] In turn, *co-design* is linked more closely to the concepts of user engagement and participatory design for the purposes of developing a new product, initiative, or service (12).

Many attempts have been made to demarcate the terms co-production and co-design, as well as involvement more broadly, but one could ask "*Does it really matter*?". A systematic review (13) of their varied definitions concluded that these terms are conceptualised in many different ways; and instead of focusing our efforts on finding a common language we should instead focus on defining the key principles and values underlying these terms; and how these could be meaningfully translated into every practice. In this Communication article, we will use the terms involvement and co-production.

Youth involvement in mental health research

Within the context of mental health research, the meaningful involvement of young people with lived or living experience in planning, delivering, and implementing research has

For peer review only - http://bmjopen.bmj.com/site/about/guidelines.xhtml

received widespread attention. Moves towards youth-led research, whereby young people as 'experts by experience' take on the role of agenda setters by defining priorities or directions of research, although slower, have been significant (14). A recent example of youth-led research (15) was undertaken by the UK Research and Innovation (UKRI)-funded Transdisciplinary Research for the Improvement of Youth Mental Public Health (TRIUMPH) mental health research network. The authors describe a UK-wide priority-setting exercise to identify public health intervention-related research to support youth mental health, offering examples of good practice in co-production, whereby young people take on the role of agents of change. The authors also reflect on the challenges of involving multiple stakeholders with different, and often competing, priorities and ensuring the voices of young people are not lost during the process of finding a shared language (15).

The significant benefits resulting from such partnerships with young people to collaboratively develop and deliver high quality mental health research (16, 17, 18) have been widely documented. For the research itself, benefits include improved data collection methods and recruitment strategies (19), more relevant research objectives enhancing study acceptability (19, 20, 21), and facilitating the translation of research findings into practice, therefore, creating real-world impact (16). For young people with lived or living experience, recent research highlights several benefits, including drawing on their own experience to help and support others, gaining a sense of achievement through the impact of their involvement, as well as personal growth and capacity building (18, 22). It is key not to ignore the potential pitfalls of co-production, such as various risks to both researchers and experts by experience (23) but also to recognise how current limitations may arise from existing contextual inequalities in academia and more broadly (11). Against the background of the historically embedded power differentials between mental health service users and professionals, noted above, the possibility of reinforcing such unequal power relations across the research's various stakeholders needs recognising (24, 25). But so too does the danger of repeating these power imbalances by shutting young people out of involvement as experts by experience.

It is also clear that for young people with lived or living experience, co-production can offer a way to make a difference, and even to reflect on one's own experiences (26, 27, 28). Then why is it that when it comes to suicide prevention research, the involvement of young people is often considered risky and unfeasible?

Youth involvement in suicide prevention research

There is, firstly, a pervasive concern about the possibility of causing distress, and even suicidal thoughts and feelings (29, 30). Such concern is commonly raised by health research ethics committees and even researchers themselves (29, 30) and relates to a deficit-based approach whereby young people with lived or living experience of self-harm or suicidal behaviour are perceived as "vulnerable", needing "protection from harm". Associated with the concern of causing harm or distress are also concerns about researchers' own competency and capacity to support potentially distressed young people, and/or their

For peer review only - http://bmjopen.bmj.com/site/about/guidelines.xhtml

families (29). This mirrors a similar concern around young people taking part as research participants too – and yet, taking part in research does not inflict harm and young people cite important benefits (31). So, why do we hold onto this concern in such a way that it acts to preclude young people from being involved in suicide prevention research as experts by experience?

The safety of the young people is of paramount importance and the risk of involvement potentially causing distress needs to be acknowledged. It may be that there are limitations to involvement posed by legitimate safeguarding, depending on the topic of the research. However, we argue that the greatest risk to young people is not being heard and included. We risk leaning too heavily on a safety discourse in a way that allows us to not collaboratively find ways to undertake co-production ethically and safely. We also run the risk of being unethical, exclusionary, and paternalistic. These specific risks that arise from, or perhaps rather are *assumed to* arise from, suicide prevention research (29, 30), show the need to be mindful of not reproducing entrenched power imbalances between experts by experience on the one hand and 'professionals' on the other.

To undertake meaningful involvement in suicide prevention research, there is a need across the team – both young people and researchers – to sit with a certain level of risk. It is key that this is acknowledged at the outset of the research and collaboratively discussed. Before this stage, however, it is also necessary to rethink the way we approach risk in drawing the team together. There is a tendency in even the most ethical and equitable involvement to nevertheless exclude people who may be, or have recently been, suicidal. Given the subject matter and the need to ensure that the voices of young people who are or have been suicidal are placed central to our research praxis, we question whether this is ethical.

The need, therefore, to reflect critically on who is 'allowed' to become involved mirrors who is 'allowed' to take part in suicide prevention research as a participant too. In both contexts, if 'we' as the researchers set the bar in a way that is too risk averse, we will perpetuate the historical power differentials in mental health research and services noted above, and ignore, exclude, and disempower people whose voices should be listened to.

We provide below an example of co-production in suicide prevention research drawn from our own research programme; highlighting opportunities for maximising the potential of partnerships between researchers and young people with lived or living experience. The case study has been informed by the second author (JM), a member of the Youth Advisory Group (YAG), at the Institute for Mental Health, University of Birmingham. The YAG consists of 18 young people aged between 18-25 with experience of or a strong interest in youth mental health, who work collaboratively with researchers to create and shape research into youth mental health including self-harm and suicide. The YAG is a diverse group of young people in terms of cultural representation, ethnic background, gender identity and sexual orientation.

For peer review only - http://bmjopen.bmj.com/site/about/guidelines.xhtml

Case study

#MyGPguide - Visiting your general practitioner: a guide for young people with lived experience of self-harm and suicidality

#MyGPguide (32) is an evidence-based resource co-designed with young people for young people with lived experience of self-harm and/or suicidal behaviour to prepare them for their consultation with their General Practitioner (GP) offering practical support and guidance on: i) What to consider before they visit their doctor, including preparing questions and booking an appointment; ii) How to manage the consultation, what their rights are with respect to confidentiality, what questions their doctor might ask them; and how to manage discussions about medication, safety planning and referral to mental health services; iii) What to do after the consultation and how their doctor can support them including signposting and accessing professional support.

Consultations about self-harm and suicidal thoughts and feelings can be challenging for both GPs and young people (33, 34). *#MyGPguide*, offers evidence-based, accessible, and practical tips to facilitate the best consultation and support for young people at-risk of suicide.

This account of the creation of #MyGPguide will highlight and reflect on various issues that have been raised when considering the feasibility, value, ethics and safeguarding of co-production in suicide prevention research.

Over a period of six months, six members from the Institute of Mental Health, University of Birmingham YAG worked in partnership with the project lead (first author) and the Institute's Youth Participation Lead (YPL) to co-design *#MyGPguide*. The role of the YPL is to facilitate meetings between researchers and the YAG; to process requests from researchers to involve the YAG in their work, and likewise help make it easy and comfortable for the YAG to be involved. With regards to safety, the YPL's role is important as they provide a constant for the YAG in their meetings with different researchers. By being a friendly face, and someone who is specifically there to facilitate the work of the YAG, the feelings that might accompany a young person in this sort of environment are effectively mitigated.

Consultations took place online due to COVID-19 restrictions at the time. The first consultation started with the YPL reminding everyone of the YAG terms of reference; and the project lead sharing key ground rules that would ensure that young people were involved in a safe, inclusive, and respectful way throughout the process. The ground rules focused on i) highlighting the importance of confidentiality (and its limits) and anonymity; ii) acknowledging and respecting different and complementary types of expertise; iii) listening respectfully to one another; iv) allowing others the time and space to share their story; v) taking turns in speaking and being mindful of dominating discussions.

The first stage of this project was an 'initial ideas' meeting which involved brainstorming and discussing initial thoughts about the content and overall direction that YAG members wanted the guide to take. This was a productive meeting and YAG members were given multiple chances to share their views. It is important to note that from the beginning and

For peer review only - http://bmjopen.bmj.com/site/about/guidelines.xhtml

throughout the whole project YAG members were never asked to share or disclose personal experiences or events that might have been relevant to the project. Instead, they were encouraged to draw upon their own experience to meaningfully inform the content of the guide. Moreover, YAG members were encouraged to contribute in different ways depending on their preference. This included sharing their views during the online consultations; writing down thoughts and emailing them to the project lead; or having a one-to-one chat with the project lead. For some YAG members, this flexible approach was invaluable as they found it difficult to share in the group some of the points that they wanted to make. These points related to their own and their friends' experience of visiting the GP and how they wanted these points conveyed in *#MyGPguide*. In response, the project lead expressed the importance of these points and their added value to *#MyGPguide*. The project lead subsequently described how these points would be incorporated in the guide ensuring that this process was carried out in a way that would accurately reflect YAG members' contribution i.e., staying true to their voice.

Following the initial brainstorming session, the first draft of *#MyGPguide* was circulated to all YAG members involved in the project and they were encouraged to read the guide and send any feedback. Feedback could be given via different ways including highlighting or using track changes on the document, or simply drafting the feedback and emailing this back to the project lead. It was clear where YAG members' comments had been included in the draft guide alongside the evidence-base.

To bring the guide to life, the project lead offered YAG members the opportunity to be involved in a few other ways. These included producing a quote relating to a specific aspect of the guide (e.g., the importance of confidentiality) to be embedded within the guide, involvement in a video that would accompany the guide, and an opportunity to be involved in a webinar to promote *#MyGPguide*. Each of these opportunities were once again accompanied by the flexibility and inclusivity which characterised the whole process of involvement.

Subsequent iterations of *#MyGPguide* were shared with the YAG, and members were always encouraged to make additional contributions and provide feedback. It was clearly demonstrated how their contributions had informed the content and design of *#MyGPguide* as well as a dissemination plan targeting diverse audiences including young people, families, primary care practitioners and schools. YAG members were kept informed throughout the project about timelines, milestones including the release of *#MyGPguide*; and supported to take part in dissemination and outreach activities to promote the guide.

Throughout the process, YAG members were never asked to reveal personal information, and any that was given was given voluntarily, without prompt and in line with the YAG terms of reference. The discussions, either as part of the group or one-to-one, were equitable and balanced. The way young people's contributions influenced *#MyGPguide* was clearly demonstrated; and where feedback had not been taken on board the rationale was provided. For example, although important, some points and ideas might not have fit the form the guide was aiming to take, could not offer additional value, or did not speak to the target audience.

For peer review only - http://bmjopen.bmj.com/site/about/guidelines.xhtml

At no point were YAG members made to feel like their contribution would make or break the guide, and they were reassured that they could withdraw at any time. Crucially, though, YAG members were consistently reminded of how their involvement and any contribution they did make was important and valued.

Furthermore, the flexibility described in this account was one of the most central themes that ensured safety in the involvement of YAG. Although extremely important, safety does not simply refer to the idea of keeping someone safe from physical or psychological harm. The term safety within the context of suicide prevention research should also be taken to mean how comfortable and able a young person is to fully draw upon their experience to inform the research they are involved in. The flexibility of involvement here was crucial to maintaining a comfortable environment. Indeed, if young people do not feel safe or comfortable, they will not be able to fully apply their experiences and expertise or they will avoid participating altogether. It is therefore in both the young persons and the researchers' interests to ensure safety during involvement.

Key lessons for youth involvement in suicide prevention research

Young people were at the forefront of designing and disseminating *#MyGPguide*. Using the case study above and our previous research experience and experiential knowledge, we draw together key lessons underlying the optimal involvement of young people in suicide-related research.

Firstly, having clearly defined and mutually agreed terms of reference and ground rules helps to generate and sustain inclusivity throughout the process of involving young people as experts by experience in suicide prevention research. As Clarke et al (35) highlighted, inclusivity is not simply about bringing together a group of stakeholders to share and exchange knowledge and views. Inclusivity is about generating and sustaining mutually agreed goals; sharing the same vision, or, as Clarke et al[35] described it "*a shared sense of identity and solidarity*".

Secondly, consulting with young people about potential ethical considerations, safety issues or stressors before and during co-production and putting in place safety protocols tailored to young people's needs can ensure their safe involvement. Such protocols could take the form of a wellness or safety plan where researchers and young people work together to identify potential stressors, strategies that the young person can use when they are feeling upset, a support person the researchers could contact if the young person wishes so. Having a one-to-one debriefing session with young people also offers them the opportunity to highlight things that worked well for them during a co-production session and things that could be improved in future sessions. Examples of good practice using wellness plans when involving young people in suicide prevention research can be used as a guide (36, 37). A risk mitigation strategy when conducting participatory modelling workshops with young people with lived or living experience of self-harm or suicidal behaviour has also been published and can be used or adapted by other researchers in the field (37). The first evidence-based guidelines for the safe and effective involvement of young people with lived and living experience in suicide research have recently been published and provide an invaluable resource for researchers (38). We cannot eliminate risk, but we can put in place robust

For peer review only - http://bmjopen.bmj.com/site/about/guidelines.xhtml

processes that help researchers mitigate risk. It is crucial to recognise that young people can draw on their own experiences to inform research (and, through this, practice and policy) without necessarily having to disclose or share those experiences. It is important to set the parameters of sharing at the outset of any research, both creating a safe space to do so and also emphasising that this is not an expectation. Having a dedicated youth participation lead to support both young people and researchers during this process in a safe, productive, and ethically sound way is key.

Thirdly, access to appropriate training, supervision and reflective practice for researchers working in “emotionally demanding research”, including undertaking co-production with young people with lived experience, is important in developing the knowledge and skills to mitigate potential risk issues but also in maintaining their own wellbeing (39).

Finally, setting a culture of open, transparent, and reciprocal communication between young people and researchers is key to the establishment of an equal relationship among all those involved in the co-production and the continual challenging of hierarchies. The principle of transparent communication has consistently been highlighted by different participatory research approaches as key in fostering authenticity, collaboration and empowerment (40, 41). Co-production should be a professional relationship between young people and researchers and, as in any other type of professional relationship, there should be clearly defined roles, clear objectives, and an expectation of being listened to and valued.

It is our ethical and moral imperative, as researchers, to create a safe and inclusive environment that would empower the involvement of young people with lived or living experience of self-harm and suicidal behaviour in research that affects them, their care and quality of life. Progress in the field of suicide prevention cannot be achieved without the meaningful involvement of young people.

For peer review only

Contributors

MM, AL and JM conceptualised the paper. MM wrote the first draft of the manuscript. All authors were involved in further revisions and writing of the final version.

Competing interests

None declared. *#MyGPguide* is a non-commercial product for which the authors receive no royalties.

Funding

While working on this Communication article, MM was funded by the European Union H2020-EU.1.3.2. MSCA-IF-GF—Global Fellowships (grant agreement ID: 101026065). JM was funded by the Institute for Mental Health, University of Birmingham.

REFERENCES

1. Millar, S.L., Chambers, M. and Giles, M. (2016), Service user involvement in mental health care: an evolutionary concept analysis. *Health Expect* 2016; **19**: 209-221.

2. Fenton, S-J., Carr, S., Boyden, L. Molloy, J., Moyse, H., Rathore, I., Skinner, B., Tresadern, C., Virgo, H., Willams, B. and Campbell, N. Valuing Youth Involvement in Mental Health Service Design and Delivery In Theodosiou, L., Knightsmith, P., Paula, P. and Bailey, S. (eds.) Children and Young People's Mental Health London: Pavilion pp. 163-178, 2020

3. INVOLVE. Briefing Notes for Researchers: Public Involvement in NHS, Public Health and Social Care Research, 2012 (page 7).

4. Henwood M. Involvement of service users in social care research. Disability, 2007 https://www.communitycare.co.uk/2007/09/12/involvement-of-service-users-in-social-care-research/ (Accessed 26 May 2023)

5. Lignou S, Capitao L, Hamer-Hunt J.M., Singh I. Co-Production: An Ethical Model for Mental Health Research? *Am J Bioeth* 2019; **19**:8, 49-51
6. Rose, D. Patient and public involvement in health research: Ethical imperative and/or radical challenge? *J Health Psychol* 2014; **19**: 1, 149-158

7. Ostrom E, Parks RB. Suburban police departments: Too many and too small? Department of Political Science, Indiana University; 1973.

8. Stephens L., Josh Ryan-Collins J., Boyle E.D. Co-production. A Manifesto for growing the core economy. New Economics Foundation. London, 2008 https://neweconomics.org/uploads/files/5abec531b2a775dc8d_qjm6bqzpt.pdf (Accessed 26 May 2023)

9. Coote A. Claiming the health dividend: unlocking the benefits of NHS spending. London: King's Fund, 2002 Claiming the Health Dividend: Unlocking the benefits of NHS spending, Summary - Anna Coote (ed) - The King's Fund, 1st May 2002 (kingsfund.org.uk) (Accessed 26 May 2023)

10. Needham C., Carr S. SCIE research briefing 31: co-production: an emerging evidence base for adult social care transformation. Social Care Institute for Excellence, London, 2019 https://lx.iriss.org.uk/sites/default/files/resources/briefing31.pdf (Accessed 26 May 2023)

11. Williams, O., Sarre, S., Papoulias, Knowles S., Robert G., Beresford P., Rose D., Carr S., Kaur M., Palmer V. Lost in the shadows : reflections on the dark side of co-production. *Health Res Policy Sys* 2020; **18**:1, 43

12. Vargas C, Whelan J, Brimblecombe J, Allender S. Co-creation, co-design and co-production for public health: a perspective on definitions and distinctions. *Public Health Res* Pract 2022; **32**:2, e3222211.
13. Masterson, D, Areskoug Josefsson, K, Robert, G, Nylander, E, Kjellström, S. Mapping definitions of co-production and co-design in health and social care: a systematic scoping review providing lessons for the future. *Health Expect* 2022; **25**: 902- 913

For peer review only - http://bmjopen.bmj.com/site/about/guidelines.xhtml

14. Wilson, O., Daxenberger, L., Dieudonne, L., Eustace, J., Hanard, A., Krishnamurthi, A., Quigley, P., and Vergou, A. (2020). A rapid evidence review of young people's involvement in health research. London: Wellcome

15. Taylor, A., McMellon, C., French, T. MacLachlan A. (2022). Defining research priorities for youth public mental health: reflections on a coproduction approach to transdisciplinary working. *Health Res Policy Sys* 2022; **20**: 72

16. Ennis L, Wykes T. Impact of patient involvement in mental health research: longitudinal study. *Br J Psychiatry* 2013; **203**:5, 381-386.

17. Staley K. A series of case studies illustrating the impact of service user and care involvement on research. London, UK: Mental Health Research Network; 2013

18. Ungar, M., McGrath, P., Black, D., Sketris, I., Whitman, S., & Liebenberg, L. Contribution of participatory action research to knowledge mobilization in mental health services for children and families. *Qual Soc Work* 2015; **14**(5): 599-615

19. Pavarini, G., Lorimer, J., Manzini, A., Goundrey-Smith, A., & I, S. Co-producing research with youth: The NeurOx young people's advisory group model. *Health Expect* 2019; **22**: 743–751

20. Garinger, C. A., Reynolds, K. A., Walker, J. R., Firsten-Kaufman, E., Raimundo, A. S., Fogarty, P. C., . . . Research Group, M. M. Mobilizing Minds: Integrated knowledge translation and youth engagement in the development of mental health information resources. *Gateways: International Journal of Community Research and Engagement* 2016; **9**(1): 172

21. Gishawn, A. M., Tamar, M., Benjamin Byrd, I., Jahon, J., & Darius, T. Utilizing Community-Based Participatory Research to Adapt a Mental Health Intervention for African American Emerging Adults. *Prog Community Health Partnersh* 2010; **4**(2): 131-140.

22. Watson, R, Burgess, L, Sellars, E, et al. A qualitative study exploring the benefits of involving young people in mental health research. *Health Expect* 2023; **26**: 1491-1504

23. Oliver K, Kothari A, Mays N. The dark side of coproduction: do the costs outweigh the benefits for health research? *Health Res Policy Syst* 2019; **17**:1. 33.

24. Carr S. Can Co-production Really Transform UK Mental Health Services? Mad in America, 2016. https://www.madinamerica.com/2016/08/can-co-production-really-transform-uk-mental-health-services/ (Accessed 26 May 2023)

25. Daya, I., Hamilton, B. and Roper, C. Authentic engagement: A conceptual model for welcoming diverse and challenging consumer and survivor views in mental health research, policy, and practice. *Int J Mental Health Nurs* 2020; **29**:2, 299-311.

26. Awenat YF, Moore C, Gooding PA, Ulph F, Mirza A, Pratt D. Improving the quality of prison research: a qualitative study of ex-offender service user involvement in prison suicide prevention research. *Health Expect* 2018; **21**:1, 100-109.

For peer review only - http://bmjopen.bmj.com/site/about/guidelines.xhtml

27. MacLean S, MacKie C, Hatcher S. Involving people with lived experience in research on suicide prevention. *CMAJ* 2018; **190**(Suppl), S13-S14.

28. Dold, C. J., & Chapman, R. A. Hearing a voice: Results of a participatory action research study. *JCFS* 2012; **21**: 512-519

29. Lakeman, R., & FitzGerald, M. (2009). The ethics of suicide research: The views of ethics committee members. *CRISIS* 2009; **30**(1): 13–19

30. Wadman R, Williams AJ, Brown K, Nielsen E. Supported and valued? A survey of early career researchers' experiences and perceptions of youth and adult involvement in mental health, self-harm and suicide research. *Res Involv Engagem*. 2019; **29**: 5:16

31. Lockwood, J., Townsend, E., Royes, L. *et al.* What do young adolescents think about taking part in longitudinal self-harm research? Findings from a school-based study. *Child Adolesc Psychiatry Ment Health* 2018; **12**: 23

32. Michail M., Rowmell H., Mitchell L., Morgan J., Rathore I., Reid K., Tresadern C. et al. *#MyGPguide* - Visiting your General Practitioner: A guide for young people with lived experience of self-harm and suicidality. Birmingham: Institute for Mental Health, School of Psychology, University of Birmingham, 2021 https://doi.org/10.6084/m9.figshare.17069660.v1 (Accessed 26 May 2023)

33. Michail M, Tait L. Exploring general practitioners' views and experiences on suicide risk assessment and management of young people in primary care: a qualitative study in the UK. *BMJ Open* 2016; **6**: e009654

34. Farr J, Surtees ADR, Richardson H, Michail M. Exploring the processes involved in seeking help from a General Practitioner for young people who have been at risk of suicide. *Int J Environ Res Public Health* 2021; **18**:4, 2120.

35. Clarke, J, Waring, J, Timmons, S. The challenge of inclusive coproduction: The importance of situated rituals and emotional inclusivity in the coproduction of health research projects. *Soc Policy Admin* 2019; **53**: 233– 248

36. Thorn P, Hill NT, Lamblin M, Teh Z, Battersby-Coulter R, Rice S, Bendall S, Gibson KL, Finlay SM, Blandon R, de Souza L, West A, Cooksey A, Sciglitano J, Goodrich S, Robinson J. Developing a Suicide Prevention Social Media Campaign With Young People (The #Chatsafe Project): Co-Design Approach. *JMIR Ment Health* 2020;**7**(5):e17520

37. Michail M, Robinson J, Witt K, et al. Which programmes and policies across health and community settings will generate the most significant impacts for youth suicide prevention in Australia and the UK? Protocol for a systems modelling and simulation study. *BMJ Open* 2023;**13**: e071111

38. Webb M, Cooper C, Hemming H, Dalton A, Unity E, Simmons M, Bendall S, Robinson J. Guidelines for involving young people with lived and living experience of suicide in suicide research. Melbourne: Orygen, 2023 https://www.orygen.org.au/Training/Resources/Self-harm-and-suicide-prevention/Guidelines/Suicide-research/Guidelines-for-young-people-with-living-or-lived-e (Accessed 18 September 2023)

39. Burrell, A., Costello, B., Hobson, W. et al. Being prepared for emotionally demanding research. *Commun Psychol* 2023; **1**: 9

40. Kennedy, Heather K., "Disrupting Adultism: Practices That Enable or Constrain Intergroup Contact Between Youth and Adults" (2019). Electronic Theses and

For peer review only - http://bmjopen.bmj.com/site/about/guidelines.xhtml

Dissertations. 1667. https://digitalcommons.du.edu/etd/1667 (Accessed 18 September 2023)

41. Doucet M, Pratt H, Dzhenganin M, Read J. Nothing About Us Without Us: Using Participatory Action Research (PAR) and arts-based methods as empowerment and social justice tools in doing research with youth 'aging out' of care. *Child Abuse Negl.* **2022**;**130**(Pt 3):1053-58.

For peer review only

For peer review only - http://bmjopen.bmj.com/site/about/guidelines.xhtml
